# Differences between pre-existing type and de novo type left convex thoracolumbar / lumbar scoliosis

**DOI:** 10.1186/1748-7161-10-S2-S6

**Published:** 2015-02-11

**Authors:** Takahiro Iida, Yasumasa Ohyama, Jyunya Katayanagi, Akihisa Ato, Ken Mine, Kazuyuki Matsumoto, Hirokazu Furukawa, Takashi Tomura, Satoru Ozeki

**Affiliations:** 1 Department of Orthopaedic Surgery, Dokkyo Medical University Koshigaya Hospital, Minami-Koshigaya, Koshigaya, Saitama, Japan

## Abstract

**Background:**

Lenke 5C type adolescent idiopathic scoliosis (AIS) with a Cobb angle of over 30 degrees has high risk of progression. The need for corrective surgeries for degenerative lumbar scoliosis has been increasing these days and some of those cases are pre-existing type scoliosis. However, it is said to be difficult to differentiate pre-existing type scoliosis from de novo type scoliosis. The purpose of this study is to analyze the relevant X ray metrics of degenerative lumbar scoliosis and to discover differences between pre-existing and de novo type scoliosis.

**Methods:**

Of 54 consecutive patients who were diagnosed as candidates for corrective surgery for left convex thoracolumbar / lumbar scoliosis since December 2008, 19 patients over age 50 were included in this study. The average age was 60 years old (50-80 years old). All patients were female. Coronal and sagittal parameters were contrasted between two groups divided according to the existence of scoliosis in their adolescence; clear (AIS) and unclear (de novo).

**Results:**

Eleven were AIS, and 8 were de novo. The average age was 54.0 years old for AIS and 67.4 for de novo (p<0.05). Cobb angles (69°, 49°) and L4 tilt (30°, 22°) were found to be significantly greater in AIS. Nash-Moe rotation assessment showed that rotational deformity was greater in AIS type than in de novo type. Lumbar lordosis (28°, 32°), thoracolumbar kyphosis (24°, 12°), sagittal vertical axis (37mm, 58mm), and pelvic incidence (51°, 60°) showed no significant difference between the groups, however, pelvic tilt (24°, 33°) showed significant difference.

**Conclusions:**

Among patients over 50 with degenerative thoracolumbar / lumbar scoliosis, those with pre-existing type scoliosis were found to have greater Cobb angle, greater L4 tilt, greater rotational deformity, less pelvic tilt, and were candidates for surgery at a younger age than those with de novo type scoliosis. In other words, those with de novo type scoliosis have less coronal deformity and worse sagittal pelvic alignment than those with pre-existing type scoliosis and are not considered candidates for surgery until a more advanced age. This study demonstrates some differences between pre-existing and de novo type scoliosis, contrasts the natural history of the two types of candidates for thoracolumbar / lumbar scoliosis surgery, and suggests the importance of performing surgery for Lenke 5C type adolescent idiopathic scoliosis at a younger age.

## Background

Corrective surgeries for degenerative lumbar scoliosis are on the rise lately. Degenerative lumbar scoliosis consists of various pathologies, such as newly developed scoliosis after advanced age (de novo) and secondary degenerative idiopathic scoliosis (pre-existing). Differences have been pointed out between de novo and pre-existing scoliosis, however, it can be difficult to differentiate them in advanced stage. The purpose of this study is to analyze the relevant X ray metrics of degenerative lumbar scoliosis and to discover differences between pre-existing and de novo type scoliosis

## Methods

Of 54 consecutive patients who were diagnosed as candidates for corrective surgery for left convex thoracolumbar / lumbar scoliosis since December 2008, 19 patients over age 50 were included in this study. The average age was 60 years old (50-80 years old). Characteristics of the curve, coronal and sagittal parameters were investigated. Characteristics of the curve were apex of the curve, number of involved vertebrae (NOV), upper end vertebra (UEV), lower end vertebra (LEV), lateral slip, and Relevant X ray metrics were Cobb angle, trunk shift, L4 tilt, lumbar lordosis, thoracolumbar kyphosis, sagittal vertical axis (SVA), pelvic incidence (PI), pelvic tilt (PT), Nash-Moe rotation assessment, and CT rotation assessment performed by Aaro-Dahlborn method. These characteristics were contrasted between two groups divided according to the existence of scoliosis in their adolescence; clear (AIS) and unclear (de novo). Statistical analysis was performed using Students’ t-test using Statflex V6 (YUMIT, Japan). Statistical significance was based on a P-value less than 0.05. This study was approved by the Ethics Committee of Dokkyo Medical University Koshigaya Hospital. Written informed consent was obtained from the patients.

## Results

**Figure 1:**
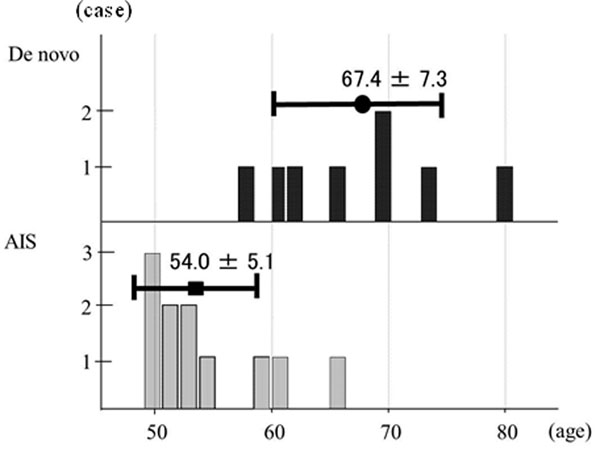
**Age distribution of both groups**. The average age was significantly higher for de novo than for AIS.

Eleven were AIS, and 8 were de novo. The average age was 54.0±5.1 years old for AIS and 67.4±7.3 for de novo, with significant difference (Figure 1).

### Characteristics of the curve

UEV and LEV were not significantly different. NOV averaged 5.6 for both groups, without any significant difference. Lateral slipping was found in 100% of de novo and 90.9% of AIS cases. The apex of the coronal curve was around L1 vertebra or L1/2 disc for both de novo and AIS groups, and the kyphosis apex was around T12/L1 disc for both groups. There was dissociation of one vertebra between the two apexes for both groups. No significant differences were observed in the characteristics of the curve between de novo and AIS groups.

### Relevant X ray metrics (Table 1)

**Table 1:** Results of X ray metrics

	De novo type	Pre-existing type	statistics
Lateral slipping	100%	90.9%	ns

Cobb angle	48.9 ±12.8	69.1 ±14.9	P<0.05

L4 tilt	21.6 ±4.5	30.0 ±7.2	P<0.05

Trunk shift	11.4 ±30.7	3.5 ±13.2	ns

Lumbar lordosis	31.9 ±13.4	27.6 ±16.8	ns

Thoracolumbar kyphosis	12.0 ±13.7	23.7 ±14.5	ns

SVA	57.8 ±38.6	36.5 ±20.5	ns

PI	59.8 ±12.4	51.0 ± 9.5	ns

PT	33.3 ±8.4	23.5 ±8.7	P<0.05

Aaro-Dahlborn	23.6 ±9.0	41.2 ±12.1	P<0.05

Investigated X ray metrics were shown for both groups with statistical significance.

**Figure 2:**
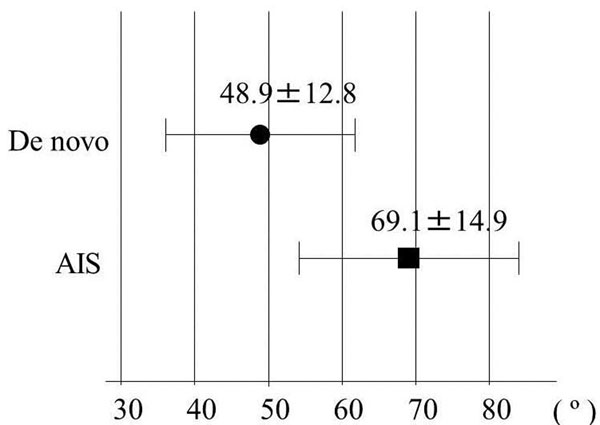
**Cobb angle**. Cobb angle was found to be significantly greater in AIS.

**Figure 3:**
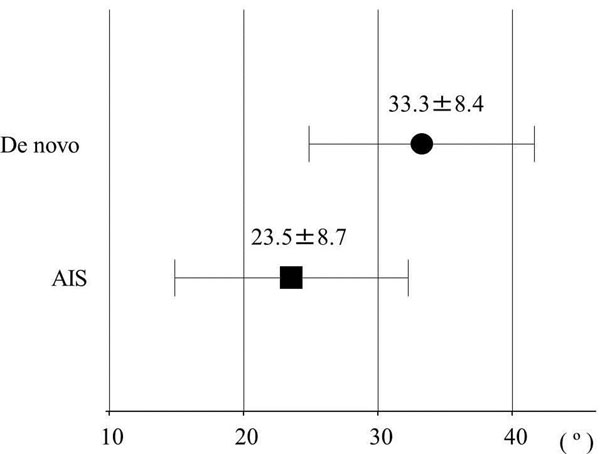
**Pelvic tilt**. Pelvic tilt showed significantly larger in De novo.

Cobb angle was 48.9±12.8 º for de novo and 69.1±14.9 º for AIS (Figure 2). L4 tilt was 21.6±4.5 º and 30.0±7.2 º respectively. Cobb angle and L4 tilt were found to be significantly greater in AIS. Trunk shift was 11.4±30.7 mm and 3.5±13.2 mm respectively without any significant difference. Lumbar lordosis (31.9±13.4 º, 27.6±16.8 º), thoracolumbar kyphosis (12.0±13.7 º, 23.7±14.5 º), SVA (57.8±38.6 mm, 36.5±20.5 mm), and PI (59.8±12.4 º, 51.0±9.5 º) showed no significant differences between the groups. PT was 33.3±8.4 º for de novo and 23.5±8.7 º for AIS, showing significantly larger in de novo (Figure 3). The Nash-Moe rotation assessment showed greater deformity in the AIS group than in the de novo group (Grade II: 5, Grade III: 3, Grade IV: 0 for de novo, 2, 4, 5, respectively for AIS). The Aaro-Dalborn rotational assessment was 23.6±9.0 º for de novo and 41.2±12.1 º for AIS also indicating rotational deformity was greater in AIS group.

## Discussion

Aebi reported that de novo type scoliosis presents less severe frontal curve, flat back or lumbar kyphosis, shorter curve, and a more prevalent stenosis than pre-existing type scoliosis [1]. Cho et al. reported that de novo type scoliosis has a higher average age, more obvious degenerative change in the vertebral body and discs, no compensatory curve in the thoracic vertebra, limited rotational deformity at the apex of the curve, lateral subluxation, sagittal imbalance more commonly, and mild Cobb angle which is generally bellow 40º [2]. Grubb et al. reported that the average Cobb angle in de novo type was 28º, while it was 52º in pre-existing type [3]. Kobayashi et al. analyzed their prospective cohort study that de novo lumbar scoliosis of 10-18º were found in 22 patients (36.7%) among 60 patients without scoliosis aged 50-84 years old who followed up for 12 years [4]. It means that it takes time for de novo scoliosis to be a large curve, resulted in not considered candidates for surgery until a more advanced age. These reports are consistent with this study.

Differences from previous reports in this study were lateral slip which was observed in almost all patients of both types of scoliosis, decreased lumbar lordosis or thoracolumbar kyphosis observed as same degree in both groups, and higher PT found in de novo type than pre-existing type whereas no significant difference seen in SVA. In pre-existing type scoliosis, coronal deformity deteriorates into a large curve according to age with lateral slipping, and sagittal deformity also develop as thoracolumbar and / or lumbar kyphosis, however, sagittal alignment is kept within normal range without large compensatory effort. By contrast, in de novo type scoliosis, coronal deformity develops to lesser curve according to age with lateral slipping, but even where sagittal deformity does not differ from pre-existing type scoliosis (within normal SVA range), greater compensatory effort is evident, and decompensated sagittal alignment is seen in some cases.

Limitations of this study are the small number of cases, the fact that we included only candidates for surgery, and that our groups were divided according to anamnesis. This suggests the possibility that mild AIS patients were included in the de novo group due to lack of proof that such patients had no scoliosis in adolescence. Further adding to that suspicion is that the average Cobb angle in our de novo group was 48.9 º, which was not greater than that in our pre-existing group, but much greater than that in Kobayashi’s cohort study.

## Conclusions

This study demonstrates some difference between pre-existing and de novo type scoliosis, contrasts the natural history of the two types of candidates for thoracolumbar / lumbar scoliosis surgery, and suggests the importance of performing surgery for Lenke 5C type adolescent idiopathic scoliosis at a younger age.

This is the extended abstract of IRSSD 2014 program book [5].

## Competing interests

The authors declare that we have no competing interests.

## Authors' contributions

TI conceived of the study, and participated in acquisition of the data, and drafting the manuscript. YO participated in acquisition of data. JK performed the statistical analysis. KM participated in analysis and interpretation of data. HF participated in analysis and interpretation of data and coordination to draft the manuscript. TT participated in revising manuscript. SO conceived of the study and revised the manuscript. All authors read and approved the final manuscript

## Declarations

Publication charges for this collection were funded by IRSSD 2014 Sapporo.

This article has been published as part of *Scoliosis* Volume 10 Supplement 2, 2015: Research into Spinal Deformities 9: Short Papers from the IRSSD 2014 Meeting. The full contents of the supplement are available online at http://www.scoliosisjournal.com/supplements/10/S2.
